# Metabolic Screening in Children with Neurodevelopmental Delay, Seizure and/or Regression

**Published:** 2017

**Authors:** Parvaneh KARIMZADEH, Mohammad Mahdi TAGHDIRI, Ezatollah ABASI, Masoud HASSANVAND AMOUZADEH, Zhila NAGHAVI, Ahad GHAZAVI, Mohammad Mahdi NASEHI, Abbas ALIPOUR

**Affiliations:** 1 Pediatric Neurology Research Center, Research Institute for Children Health, Shahid Beheshti University of Medical Sciences, Tehran, Iran; 2 Pediatric Neurology Department, Mofid Children Hospital, Faculty of Medicine, Shahid Beheshti University of Medical Sciences, Tehran, Iran; 3 Pediatric Department, Faculty of Medicine, Urmia University of Medical Sciences, Urmia, Iran; 4 Neurology and Neuroscience Research Center, Qom University of Medical Sciences, Qom, Iran; 5 Clinical Psychologist, Urmia University of Medical Sciences, Urmia, Iran; 6 Epidemiology Department, Faculty of Health, Shahid Beheshti University of Medical Sciences, Tehran, Iran

**Keywords:** Seizure, Developmental delay, Regression, Metabolic screening, Mass spectrometry

## Abstract

**Objective:**

Neurometabolic disorder is one of the important groups of diseases that prominently has presentation early infantile period. In this study, we evaluated the result of metabolic screening of the patient with seizure, developmental delay and/or regression in development, demographic disease clinical and radiological findings on admitted and outpatient visited children.

**Materials & Methods:**

Two-year retrospective review of 187 children with seizure, developmental delay and/or regression in the Mofid Children Hospital, Tehran, Iran was performed. The diagnosis was based on observation, findings of EEG and history of the patient, besides evaluation of patient milestones. The result of metabolic screening with Tandem mass spectrometry was evaluated using SPSS (ver.18.0) Statistical software.

**Results:**

Totally, 187 children with seizure, regression and/or developmental delay were evaluated by metabolic screening with tandem mass spectrometry method. The results of laboratory examination had no relationship between positive results of metabolic screening and the mentioned disease. The relations between positive results of metabolic screening and seizure, regression and/or developmental delay were not statistically meaningful.

**Conclusion:**

Positive results of metabolic screening and seizure, regression and/or developmental delay were not statistically meaningful.

## Introduction

Metabolic diseases are a group of diseases characterized by impaired function of an enzyme or vitamin necessary for the chemical reaction in the body. Enzyme deficiency leads to a lack of essential substances in the brain and a variety of metabolic diseases. Screening and identification of metabolic diseases, enzyme disorders, and newborn screening is a global necessity. Early detection and diagnosis of certain genetic, metabolic or congenital diseases can lead to a significant reduction in mortality and associated disabilities (1-10). 

Although inborn errors of metabolic diseases are rare but recurrent seizure is a common symptom of these disorders and in some resistant epileptic patients, without treatment of the underlying disease, the seizures are uncontrollable (7, 8, 11).

Metabolic disorders are not immediately symptomatic at birth and can manifest with slowly progressive encephalopathy. Nutritional problems, lethargy, vomiting, jaundice, developmental delay, sleep apnea, tachypnea, hypertonicity, ataxia, motion sickness, seizures or coma can be seen (2, 6, 8, 11, 12).

Regarding children of seizures, metabolic diseases must be considered as follows:

1. History of parental consanguinity, family history of similar disease that begins in infancy or regression of psychomotor

2. Myoclonus, and tonic spasms, unexplained persistent seizures or seizures linked to meals

3. Companionship with neurological deterioration or systemic symptoms

4. Early and progressive myoclonic epilepsy and seizures get worse with certain anticonvulsant drugs such as sodium valproate

5. EEG patterns such as burst suppression, high voltage and slow rhythmic delta waves with myoclonus and Paroxysms seen in the photic stimulation (5, 8, 13).

In this study, we tried to evaluate the result of metabolic screening of the patients with seizure, developmental delay and/or regression in development, demographic disease clinical and radiological findings in admitted and outpatient visited children in Mofid Children Hospital, Tehran, Iran from 2013-2015.

## Materials & Methods

From Mar 2013 to Mar 2015, 187 patients with seizure, developmental delay and/or regression in the Mofid Children Hospital, Tehran, Iran were evaluated. 

The diagnosis was based on observation, findings of EEG and history of the patient, and evaluation of patient milestones. The data were classified in a chart questionnaire. The data were recorded confidentially and informed consent was obtained from parents. Each person was characterized by a numerical code. 

Inclusion criteria included children with developmental delay, unusual seizures and regression that undergo mass spectrometry for metabolic screening. Exclusion criteria included patient dissatisfaction, proof of diagnosis other than developmental delay, seizures and developmental regression.

Findings were recorded using questionnaires, patient information including gender, age, family history of similar disease, the presence of family kinship, and metabolic screening.

The clinical manifestations were recorded including the early onset presentations and the signs and symptoms presented during the time of admission. The laboratory studies were all rechecked.

The result of metabolic screening with Tandem mass spectrometry was evaluated using SPSS (ver. 18.0) (Chicago, IL, USA) Statistical software.

## Results

Totally, 187 patients with seizure, developmental delay and/or regression were enrolled. The mean (SD) age of the patients was 40.93 (31.95) month, 118 patients (63.1%) of the children studied were male and 67 (36.9%) were female. Ten (5.35%) of the children surveyed had positive test results for metabolic screening.

Two children with type 1 glutaric aciduria were found. 

One of them was 6-month-old girl who presented with seizure and another was a 5-month-old boy with dystonia, seizures, developmental delay, and family history of type 1 glutaric aciduria. Both cases were confirmed by urine organic acid test. 

Two children were diagnosed as methylmalonic acidemia (MMA). One of them was 12-month-old boy who manifested with regression and developmental delay and another was 9-month-old boy with seizures, developmental delay, and regression. 

An 11-year-old girl with Carnitine Transporter Deficiency presented with seizures and moderate developmental delay in motor and speech domain. A 13-yr-old boy with seizures and moderate developmental delay with Primary Carnitine Deficiency was found. 

A 4.5-month-old boy with developmental delay was diagnosed as propionic acidemia. A 10-yr-old girl with seizures and developmental regression had Isovaleric Acidemia confirmed with investigation of urine organic acids. A 4.5-month-old girl was reported of positive test for homocystinuria and MMA. She was referred due to seizure and developmental delay. The patient’s mother had a history of homocysteinemia. An 8-yr-old girl with seizures, developmental delay, and regression underwent metabolic screening. The laboratory report raised the possibility of MMA, not confirmed by urine organic acid test. Therefore, in all analyses to examine the association between metabolic screening test results with clinical characteristics, positive result was confirmed in 9 children (Table 1). None of the patients’ clinical characteristics was significantly associated with screening test result. None of the patients’ clinical characteristics was significantly associated with Consanguinity of parents (Table 2).

None of the patients’ clinical characteristics was significantly associated with Positive family history (Table 3). None of the patients’ clinical characteristics was significantly associated with abnormal MRI (Table 4).

**Table 1:** Relationship between metabolic screening test results with clinical characteristics of the studied children

		**Total**	**Results Of Metabolic Screening**		***P*** **-value**
			**Yes** **N(%)**	**No** **N(%)**	
**Seizure**	**YES**	143	8(5.6)	135(94.4)	0.67
	**NO**	48	4(8.3)	44(91.7)	
**Developmental** **regression**	**YES**	137	5(3.6)	132(96.4)	0.24
	**NO**	32	0(0)	32(100)	
**Speech Developmental** **Delay**	**YES**	135	8(5.9)	127(94.2)	0.36
	**NO**	32	0(0)	32(100)	
**Motor** **Developmental** **Delay**	**YES**	137	9(6.6)	128(93.4)	0.21
	**NO**	39	0(0)	39(100)	

**Table 2.** Relationship between Consanguinity of parents of children with clinical features

		**Total**	**Consanguinity of parents**		***P*** **-value**
			**Yes** **N(%)**	**No** **N(%)**	
**Seizure**	**Yes**	136	73(81.1)	63(75.9)	0.4
	**No**	37	17(18.9)	20(24.1)	
**Developmental** **regression**	**Yes**	43	22(23.9)	21(25.6)	0.8
	**No**	131	70(76.1)	61(74.4)	
**Speech Developmental** **Delay**	**Yes**	124	66(76.7)	58(82.9)	0.35
	**No**	32	20(23.3)	12(17.1)	
**Motor** **Developmental** **Delay**	**Yes**	126	65(73)	61(80.3)	0.28
	**No**	39	24(27)	15(19.7)	

**Table 3:** Relationship between Positive family history of children with clinical features

		**Total**	**Positive family history**		***P*** **-value**
			**Yes** **N(%)**	**No** **N(%)**	
**Seizure**	**Yes**	134	49(83.1)	85(75.9)	0.28
	**No**	37	10(16.9)	27(24.1)	
**Developmental** **regression**	**Yes**	43	13(21.7)	30(28.8)	0.46
	**No**	129	47(78.3)	82(73.2)	
**Speech Developmental** **Delay**	**Yes**	123	40(74.1)	83(83)	0.19
	**No**	31	14(25.9)	17(17)	
**Motor** **Developmental** **Delay**	**Yes**	125	43(74.1)	82(78.1)	0.56
	**No**	38	15(25.9)	23(21.9)	

**Table 4:** Relationship between Abnormal MRI of children with clinical features

		**Total**	**Abnormal MRI**		***P*** **-value**
			**Yes**	**No**	
**Seizure**	**Yes**	117	60(80)	57(81.4)	0.82
	**No**	28	15(20)	13(18.6	
**Developmental** **regression**	**Yes**	36	17(22.4)	19(27.1)	0.5
	**No**	110	59(77.6)	51(72.9)	
**Speech Developmental** **Delay**	**Yes**	108	53(75.7)	55(87.3)	0.08
	**No**	25	17(24.3)	8(12.7)	
**Motor** **Developmental** **Delay**	**Yes**	107	52(72.2)	55(82.1)	0.17
	**No**	32	20(28.8)	12(17.9)	

## Discussion

Our study is the first one, which is reviewing results of metabolic screening of the patients with seizures, developmental delay and/or regression. In this screening, about 20 metabolic diseases were evaluated. 

Before our study, few case reports had been reported (4-6).

The mean age of the patients in our study was 40.93 months, which was consistent with previous studies (4- 6).

In this study from 143 patients who had seizures (often refractory and early onset), 8 (5.6%) had abnormal metabolic screening test results, of course, this result was not statistically meaningful (P=0.4) and less than previous studies (4, 5, 10). This difference may be attributed to the type of metabolic screening tests in previous studies (often lysosomal storage diseases).

In the evaluation of 48 patients who had developmental regression, 4 patients (8.3%) had abnormal metabolic screening test results. The statistical studies also showed no meaningful relationship (2, 4, 10). 

On the evaluation of 88 patients who had speech developmental delay, 7 cases (7.95%) were positive for metabolic screening test (P=0.15), a significant statistical relationship was not found between speech developmental delay and metabolic screening. This finding is not consistent with previous studies (2, 4, 13-17). 

On the evaluation of 128 children with motor developmental delay, 9 cases were positive for metabolic screening test. The statistical studies also showed no meaningful relationship (P=0.1). This amount was different in other studies (1-10, 13, 18-24).

All of the cases with seizures, developmental delay and regression were underwent brain MRI study which is the most common requested modality of investigation. 

This finding was consistent with previous studies (4-7, 10, 19). 

**In conclusions, **although almost all patients in this study had unusual seizures, developmental delay or developmental regression and underwent metabolic screening test, positive results of metabolic screening and seizure, regression and/or developmental delay were not statistically meaningful.
